# First person – Fatima Batool

**DOI:** 10.1242/bio.061922

**Published:** 2025-02-25

## Abstract

First Person is a series of interviews with the first authors of a selection of papers published in Biology Open, helping researchers promote themselves alongside their papers. Fatima Batool is first author on ‘ The combinatorial binding syntax of transcription factors in forebrain-specific enhancers’, published in BiO. Fatima is a PhD student in the lab of Professor Dr Amir Ali Abbasi at the National Center for Bioinformatics, Quaid-I-Azam University, Islamabad, Pakistan, decoding cis-regulatory grammar to establish a framework for discovering tissue-specific enhancers and advancing our understanding of enhancer function.


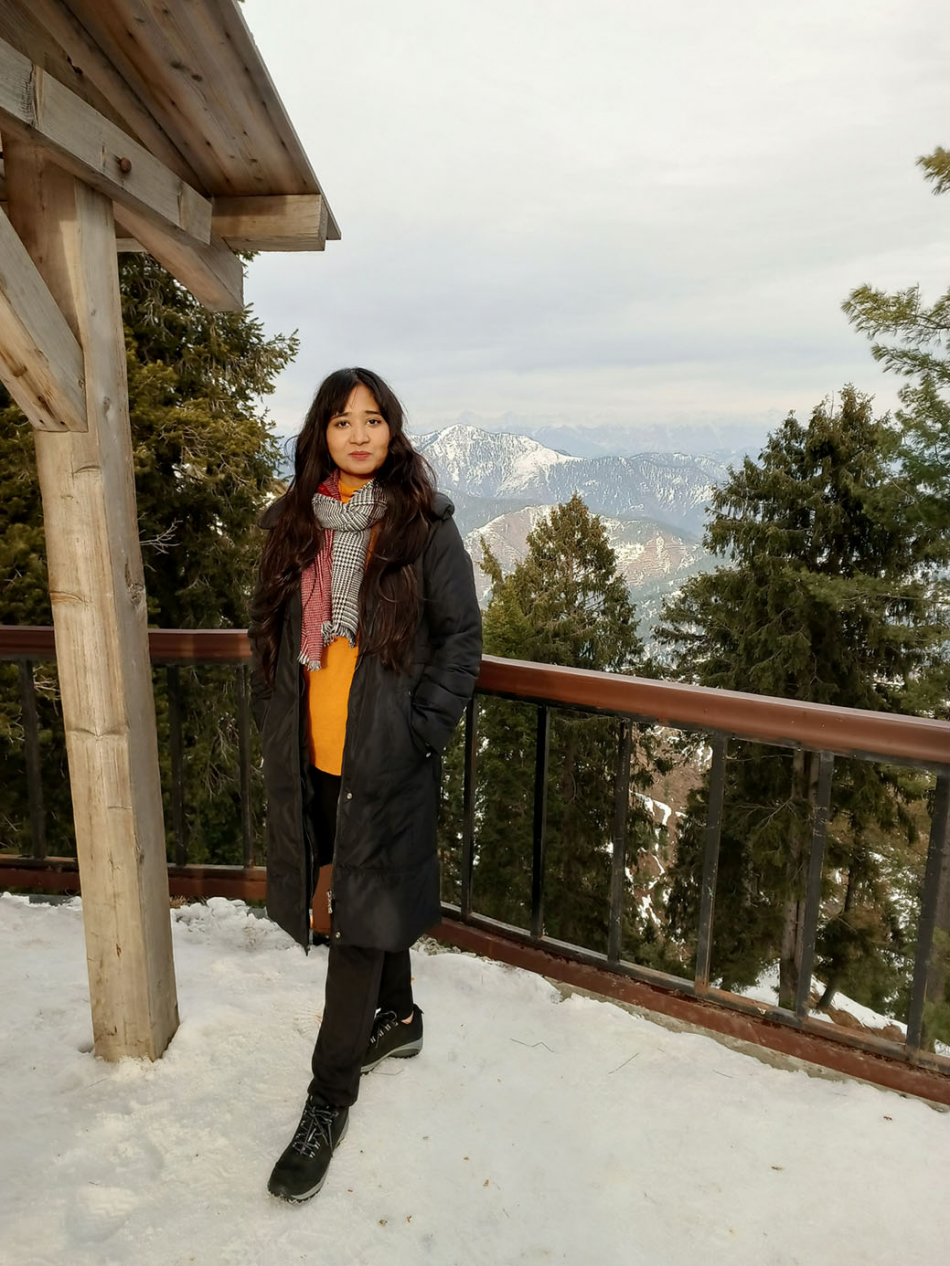


**Fatima Batool**

**Describe your scientific journey and your current research focus**

My scientific journey began with a fascination for genomics, particularly when I learned how species with highly similar genomes, like humans and chimpanzees, can show striking phenotypic and cognitive differences. These differences are largely driven by variations in gene expression, regulated by the ‘non-coding’ regions of the genome. This inspired me to focus on how non-coding DNA, specifically enhancers and their associated transcription factors, control tissue-specific gene expression in the brain. My current research seeks to decipher the language or syntax governing forebrain-specific enhancers to uncover the regulatory logic behind human brain development.

**Who or what inspired you to become a scientist?**

My inspiration to become a scientist dates back to high school, when I read about Sir Alexander Fleming's discovery of penicillin in an essay by Patrick Pringle in my English literature book. His story of curiosity, observation, and persistence sparked my fascination with biological science. Since then, it has been my passion to explore the unknown and seek answers to the many ‘whys’ that drive scientific discovery.

**How would you explain the main finding of your paper?**

Our study shows how certain DNA sequences (enhancers) work together with activating factors (transcription factors) to turn on specific genes in the developing brain. We found that these sequences follow a set of rules to make sure the right genes are activated in the human forebrain. This helps us understand how the brain develops and how problems with gene regulation might cause brain disorders.

**What are the potential implications of this finding for your field of research?**

Understanding how regulatory DNA controls gene expression in the forebrain can help us uncover the basic rules of brain development. These findings could also provide insights into developmental brain disorders, where gene regulation goes wrong, and may guide future research on designing targeted therapies.

**Figure:**
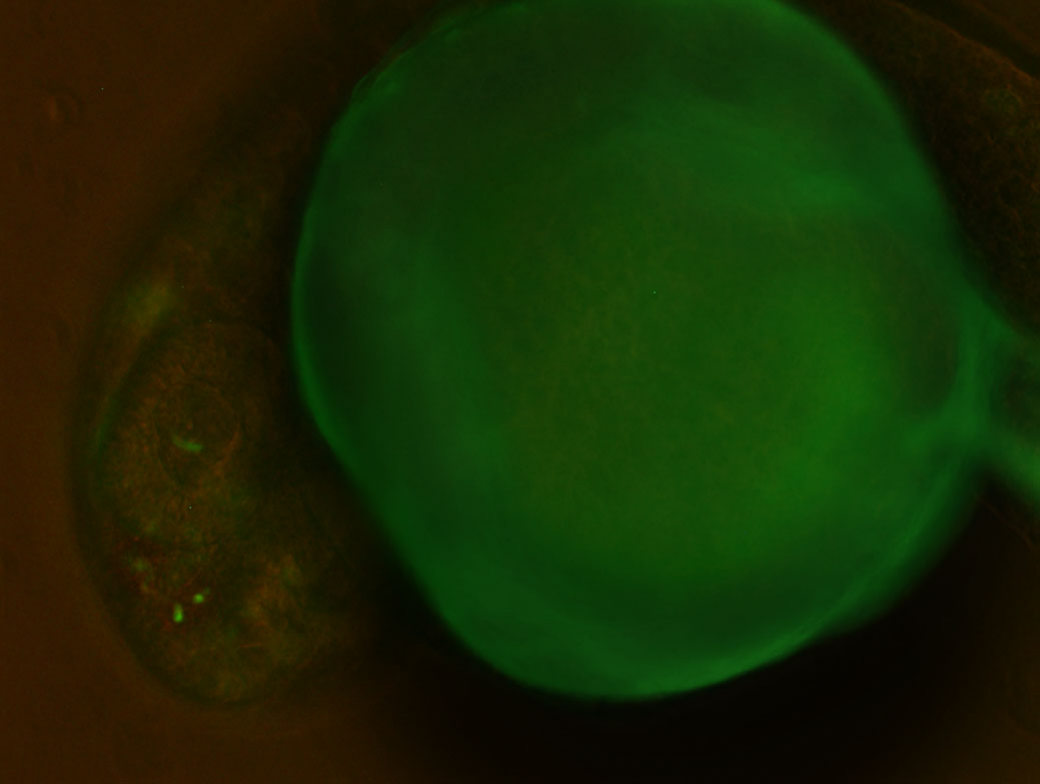
A live transgenic zebrafish embryo displaying GFP expression in the forebrain, driven by the identified regulatory enhancer, at approximately 24-48 h post-fertilisation.

**Which part of this research project was the most rewarding?**

The most rewarding part of this project was observing the *in vivo* activity of forebrain-relevant enhancers in transgenic zebrafish. Seeing the green fluorescence light up specifically in the forebrain region under the microscope was an exciting and validating moment. It was incredible to watch our predictions come to life and visually confirm the enhancer activity we had been analysing for months.

What I love most is spending time in the lab, where every experiment feels like a step closer to uncovering something new

**What do you enjoy most about being an early-career researcher?**

As an early-career researcher, I enjoy the freedom to explore different fields of science and the excitement of working on diverse, interesting projects. I have the time and energy to pursue questions that truly intrigue me and focus on finding meaningful answers. What I love most is spending time in the lab, where every experiment feels like a step closer to uncovering something new.

**What piece of advice would you give to the next generation of researchers?**

My advice to the next generation of researchers would be to approach your work with passion and curiosity, rather than seeing it as just a job or task. Be genuinely fascinated by science and stay open to learning new techniques and methods. It's important to be self-sufficient and patient, don't look for shortcuts, and understand that meaningful results take time. Embrace the journey of discovery, even when the outcomes are not immediate.

**What's next for you?**

Next, I plan to continue exploring the role of the non-coding genome in regulating gene expression and tissue development in humans. I aim to further deepen my understanding of this area, particularly how it impacts human development. My goal is to pursue postdoctoral research at a reputable institution, where I can broaden my knowledge of the human genome and contribute to advancing the field.
